# Reliability of CT‐based texture features: Phantom study

**DOI:** 10.1002/acm2.12666

**Published:** 2019-06-20

**Authors:** Bino A. Varghese, Darryl Hwang, Steven Y. Cen, Joshua Levy, Derek Liu, Christopher Lau, Marielena Rivas, Bhushan Desai, David J. Goodenough, Vinay A. Duddalwar

**Affiliations:** ^1^ Dept. of Radiology Univ. of Southern California Los Angeles CA USA; ^2^ The Phantom Laboratory Greenwich NY USA; ^3^ Department of Radiology George Washington University Washington DC USA

**Keywords:** computed tomography, imaging, phantom study, reliability, texture analysis

## Abstract

**Objective:**

To determine the intra‐, inter‐ and test‐retest variability of CT‐based texture analysis (CTTA) metrics.

**Materials and methods:**

In this study, we conducted a series of CT imaging experiments using a texture phantom to evaluate the performance of a CTTA panel on routine abdominal imaging protocols. The phantom comprises of three different regions with various textures found in tumors. The phantom was scanned on two CT scanners viz. the Philips Brilliance 64 CT and Toshiba Aquilion Prime 160 CT scanners. The intra‐scanner variability of the CTTA metrics was evaluated across imaging parameters such as slice thickness, field of view, post‐reconstruction filtering, tube voltage, and tube current. For each scanner and scanning parameter combination, we evaluated the performance of eight different types of texture quantification techniques on a predetermined region of interest (ROI) within the phantom image using 235 different texture metrics. We conducted the repeatability (test‐retest) and robustness (intra‐scanner) test on both the scanners and the reproducibility test was conducted by comparing the inter‐scanner differences in the repeatability and robustness to identify reliable CTTA metrics. Reliable metrics are those metrics that are repeatable, reproducible and robust.

**Results:**

As expected, the robustness, repeatability and reproducibility of CTTA metrics are variably sensitive to various scanner and scanning parameters. Entropy of Fast Fourier Transform‐based texture metrics was overall most reliable across the two scanners and scanning conditions. Post‐processing techniques that reduce image noise while preserving the underlying edges associated with true anatomy or pathology bring about significant differences in radiomic reliability compared to when they were not used.

**Conclusion:**

Following large‐scale validation, identification of reliable CTTA metrics can aid in conducting large‐scale multicenter CTTA analysis using sample sets acquired using different imaging protocols, scanners etc.

## 1 INTRODUCTION

With the technological advancements in medical imaging, radiomics, defined as the high‐throughput extraction of quantitative features from routine medical images to create a mineable database of imaging metrics, has emerged as a promising tool for decision support.^1^, ^2^ Radiomic metrics assessing tumor shape, nonuniform grayscale appearance (texture) which are difficult to assess visually have been reported to provide information regarding tumor diagnosis, prognosis, and treatment response.^1^, ^2^, ^3^, ^4^, ^5^, ^6^, ^7^ In spite of various benefits within the clinical workflow such as objective whole tumor assessment at no additional imaging cost and longitudinal disease monitoring, limitations with the standardization of the method reduce its reliability, particularly in multicenter studies.^8^, ^9^, ^10^, ^11^, ^12^, ^13^, ^14^, ^15^, ^16^

Typical radiomic workflows comprise of four stages: image acquisition, region of interest (ROI) segmentation, feature extraction and statistical analysis.^6^ Each of these four stages can be implemented using a variety of approaches and techniques. Currently, there is no consensus regarding a standardized implementation of the radiomics workflow, thereby hampering efforts to reproduce results.

Previous studies assessing the reproducibility of radiomic metrics conclude that radiomic metrics are not equally sensitive or insensitive to changes in scanning protocols or CT scanners. Therefore, careful consideration of the type of radiomic metrics is warranted based on the clinical application particularly in multicenter studies to avoid the chances of false discovery.^16^

A recent systematic review conducted by Traverso et al.^16^ assessing the repeatability and reproducibility of radiomics identified that most current studies report high‐risk of type I error, thereby increasing the chances of false discovery.^17^ In addition, the use of correlated metrics within the radiomics panel increases the chances of false associations with high significance.^18^ One of the solutions suggested by Traverso et al.^16^ to reduce the risk of false‐positive associations in radiomic studies is to identify reproducible and repeatable radiomic metrics and use them to train predictive models of tumor behavior. To address this concern, we conducted a series of CT imaging experiments using a texture phantom on multiple scanners and scanning protocols to assess the reliability of CTTA metrics. We define CTTA reliability as a measure of intra‐scanner variability, inter‐scanner variability and test‐retest performance of the CTTA metrics. To assess the intra‐scanner variability or the “robustness” of the CTTA metrics, images of the texture phantom are obtained using a variety of imaging conditions (scanning parameters) on a given scanner and assessed using a CTTA panel. CTTA metrics that show a strong unchanging signal across the various imaging conditions are identified as “robust” CTTA metrics. To assess the test‐retest variability or the “repeatability” of the CTTA metrics, images of the texture phantom are obtained using a variety of imaging conditions on a given scanner, 15 min apart and the difference in the performance of the CTTA panel across the two time‐points is calculated. CTTA metrics that show small to no changes in their values across the various imaging conditions are identified as “repeatable” CTTA metrics. To assess the inter‐scanner variability or the “reproducibility” of the CTTA metrics, CTTA metrics with consistent performance of the robustness and repeatability were shortlisted across scanners.

Through this study, we determine which type of CTTA metric is most reliable within the limitations of the study and we provide heatmaps of metrics such as robustness, repeatability, and reproducibility showing comparative performance of the various CTTA metrics.

## 2 MATERIALS AND METHODS

### 2.1 CTTA Phantom

**Figure 1:**
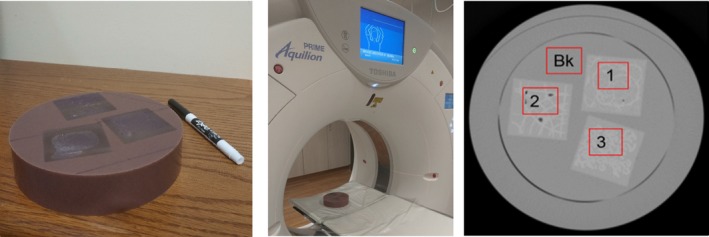
(Left) Texture phantom comprising of three texture patterns. (Middle) Phantom placement for image acquisition. (Right) Cross section of texture phantom patterns. (1), (2) and (3) are 3D printed ABS plastic with fill levels 10%, 20%, and 40%, respectively. (Bk) is a homogenous ABS material. (The window level is −500 HU with a width of 1600 HU).

Most commercially available CT phantoms are designed to be homogeneous throughout their volume; however, in real life human anatomy has variable densities inside creating texture. In our study, to evaluate the reliability of the CTTA metrics, we develop a texture phantom. The phantom comprises of three texture patterns within a homogenous background representative of textures seen in medical images (Fig. 1). The patterns were 5cm x 5cm in a 15 cm short cylinder. The phantom patterns were made using acrylonitrile butadiene styrene (ABS) plastic using 3D printing technologies and casting them into tissue density urethane. The patterns Bk, 1, 2 and 3 represent texture varying from the smoothest, that is, the background, to 10% fill, 20% fill, and 40% fill. Our intention was to create a generic phantom that could be imaged using diverse imaging protocols and scanners to identify reliable CTTA metrics. It was not our intention to create a tumor‐specific phantom as much as it was to create a phantom that covers a wide enough span of tumor textures seen in oncological CT images.

The goal of our project was to be able to reproducibly create and manipulate textures. For this, we used a 3D printer to create the texture patterns. While we tried a variety of approaches, we are currently focusing on creating reproducible geometric patterns, which could be varied in different ways to better understand how changes in patterns drive texture and its analysis. The materials selected for our tests were within the tissue density and texture range and provided targeted contrast within our texture patterns. These target Hounsfield number ranges were based on an evaluation of patient images.

### 2.2 CTTA phantom imaging

The texture phantom was scanned using a Philips Brilliance 64 CT and Toshiba Aquilion Prime 160 CT scanner. The phantom was fixed on the CT patient table for the duration of this study. The image acquisition scanning positioning for each volume was rigidly set to produce identically positioned slices, therefore obviating any need for volume registration. For the robustness assessment, 21 different settings, were tested on the Philips scanner and 16 different settings were tested on the Toshiba scanner (Table 1). For each setting, the value of only one imaging parameter was changed from the baseline scan, keeping all other variables constant. For the repeatability assessment, the CTTA results acquired from scans obtained 15 min apart on the same scanner were compared to each other. Reproducibility assessment was conducted by comparing the consistency of the robustness and repeatability signal across the two scanners. For the CTTA analysis only ROIs 1, 2, and 3 were analyzed. The post‐processing variables, i.e., IDOSE levels (1–6) on the Philips scanner and Mild/Strong on the Toshiba scanner were adjusted after all the scans. All acquisition parameters were held constant while varying the post‐processing variables, one at a time.

**Table 1:** Imaging parameters that were varied across the two scanners

Imaging parameters	Philips Brilliance 64 CT	Toshiba Aquilion Prime 160 CT
Slice thickness (mm)	1x1, 2x2, 3x3, 4x4, 5x5	1x1, 2x2, 3x3, 4x4, 5x5
FOV (mm)	125, 500	125, 500
Post‐reconstruction filtering	I‐dose (1,2,3,4,5,6)	Mild, Strong
Tube voltage (kVp)	80, 100, 140	100, 135
Tube current (mA)	40, 60, 80,100	40,60,80,100

### 2.3 Region of interest segmentation

The ROI delineation was performed using a manual segmentation technique. Three spherical ROIs were segmented in 3D using image‐rendering software (Synapse 3D, Fujifilm, Stamford CT). Some images of the phantom had air bubbles created as a result of the construction process, care was taken to exclude these regions when the analysis was performed.

Custom MATLAB (Mathworks, Natick, MA, USA) code was used to extract voxel data corresponding to the ROI. Two‐dimensional CTTA was conducted on the orientation that provided the largest diameter in the axial, coronal, or sagittal dimension. Three‐dimensional CTTA was conducted on the whole ROI volume. We used a 20‐bin gray‐level quantization. The slice thickness varied between 2 and 3 mm.

### 2.4 Image data

From the segmented ROI within the texture phantom, highlighted in Fig. 1, the CTTA metrics were extracted.

### 2.5 CTTA metrics

Texture analysis involves the study of the variation of pixel image intensity. We evaluated eight different types of texture quantification techniques on each ROI image with 235 different texture metrics. These techniques have been described in the literature^19^, ^20^, ^21^, ^22^, ^23^ (Supplementary S1: Details of CTTA metrics).

#### 2.5.1 Histogram analysis

We implemented histogram analysis which focused on the first‐order statistical analysis of texture,^19^ that is, the technique focused on assessing image intensities (Gray‐level distribution of an image), with no regard for the spatial location of the intensities. (13 features).

#### 2.5.2 Two‐dimensional and Three‐dimensional Gray‐level co‐occurrence method (GLCM) and Gray‐level difference method (GLDM) Analysis

We performed second‐order statistical analysis of texture, which included 2D‐ and 3D‐GLCM and GLDM analysis. These analyses took into account both, the pixel intensities and their inter‐relationships, thereby providing spatial information of the intensities (2nd order texture analysis) in various forms. For workflow implementation, the number of gray levels was reduced to 12‐bit, which was determined to be sufficiently accurate for the study of texture. Sixty different metrics were calculated in 2D analysis. In the 3D analysis, 20 additional directions in the z‐plane were added. (80 × 2 = 160 features).

#### 2.5.3 Two‐dimensional fourier analysis

A 512‐point fast Fourier transform (FFT) was applied to all images. Matlab® (Mathworks, Natick, MA) was used to apply the transformation.^21^ Applying the FFT algorithm, we extracted the individual frequencies, their amplitude (how much of frequency of a given type is present in the image), and phase (where in the image a given frequency is present), of the original image. FFT metrics were assessed between 10% and 90% of the maximum frequency to avoid high‐ and low‐frequency noise, which is typical for medical images. The frequency boundary was set based on maximization of the signal to noise ratio. (18 features).

#### 2.5.4 Two‐dimensional and Three‐dimensional Gray‐level run‐length method (GLRLM) analysis

We performed additional second‐order statistical analysis of texture, which included 2D‐ and 3D‐GLRLM.^22^ This analysis took into account the spatial relationship between pixels/voxels to each other by evaluating the frequency with which a given value of voxels occurs next to each other in a given direction. The 2D analysis comprised of 33 metrics and 3D analysis included 11 more metrics (44 features).

### 2.6 Statistical analysis

**Figure 2:**
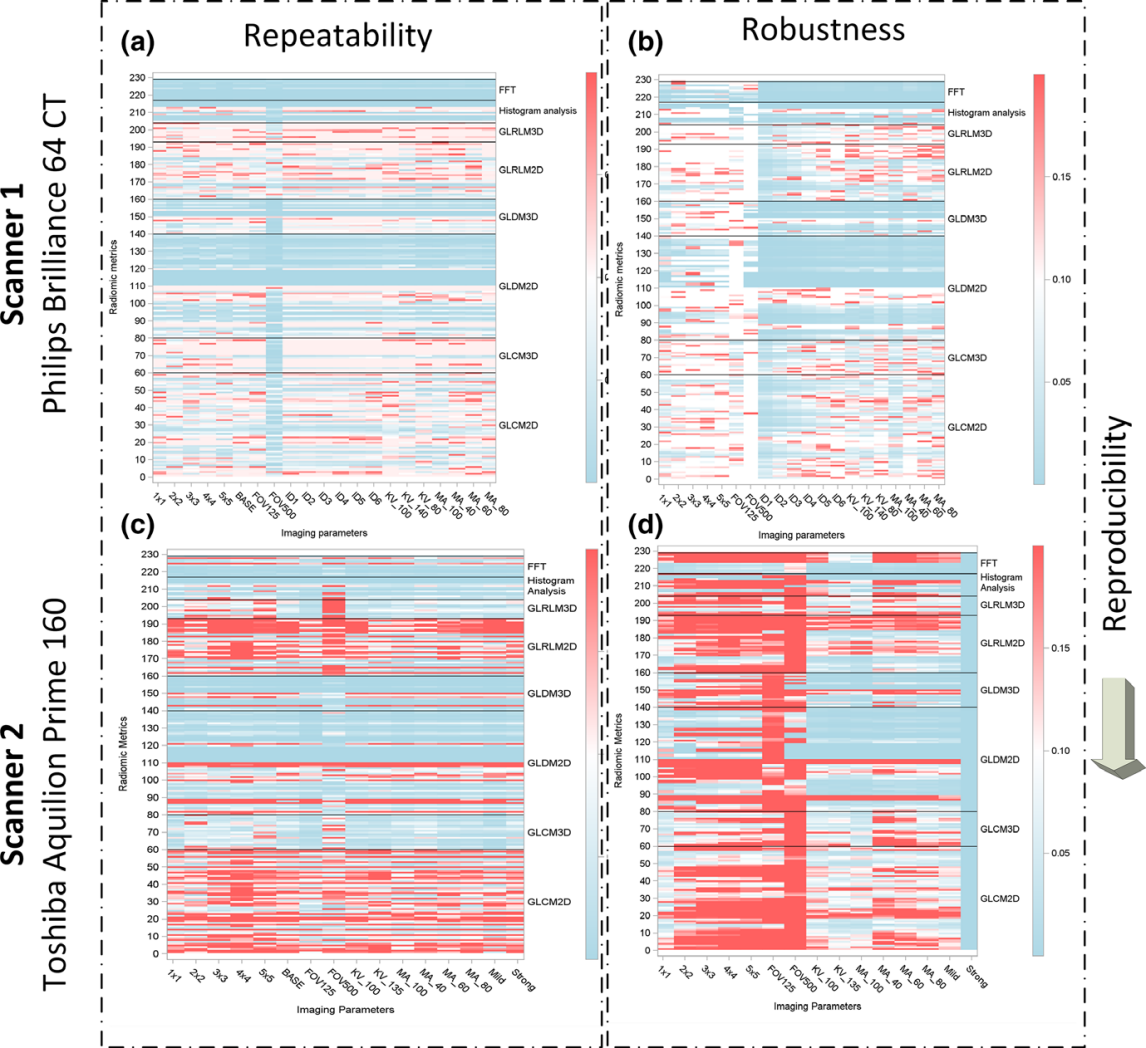
Reliability assessment of the texture metrics of the USC Radiomics panel using two different CT scanners.

For the robustness test, 21 unique image settings were tested on the Philips scanner (x‐axis of Fig. 2A) and 16 unique settings were tested on the Toshiba scanner (x‐axis of Fig. 2C). From the images acquired for each setting, three ROIs (ROI 1, 2 and 3 from Fig. 2) were segmented and analyzed using the texture panel of the USC radiomics framework. The texture panel comprised of 229 features belonging to eight subgroups of texture extraction methods (y‐axis of Fig. 2A). The repeatability test was conducted 15 min apart for all the image settings on both scanners.

For the repeatability test, the percent absolute difference between each of the radiomic metrics in the initial scan and the scan 15 min later was plotted for each scanner [Figs. 2(a) and 2(C)]. For the robustness test, the percent absolute difference between each of the radiomic metrics in the baseline scan setting and new settings (x‐axis variables) was plotted for each scanner [Figs. 2(a) and 2(C)]. The variables were changed one at a time with respect to the baseline scan settings. The percent absolute difference in the radiomic metrics in the repeatability and robustness study has been presented as a heatmap ranging from 0% (blue) to 20% (red) variation.

In the repeatability heatmap (Fig. 2), a solid horizontal blue band represents good repeatability across these acquisition parameters (here, <5% absolute difference between various settings). In the robustness heatmap (Fig. 2), a solid horizontal blue band represents good robustness across these acquisition parameters (here, <5% difference between various settings).

## 3 RESULTS

Our results indicate that the reliability of radiomics metrics is dependent on the scanner and scanning settings.

### 3.1 Repeatability

The percentages of repeatable CTTA according to the test‐retest analysis, on the Philips scanner is 97.08% (4771 of 4914 CTTA metrics) when the cutoff value of 0.15 is chosen for the percent absolute difference. A similar analysis on the Toshiba scanner showed reduced repeatability of 74.01% (2771 of 3744 CTTA metrics). In general, second‐order texture metrics such as GLCM, GLDM, and GLRLM show poor repeatability in both the Philips and Toshiba scanners. Three‐dimensional versions of these metrics show improved repeatability on the Toshiba scanner in comparison to the Philips scanner. Consistently, FFT metrics analysis metrics show good repeatability (blue bands in the repeatability map next to FFT, Metric 218 220 224 and 225).

### 3.2 Robustness

Regarding the influence of the modification of the CT acquisition parameters on the reproducibility of RFs, results varied depending on the scanner and imaging parameters. When robustness was evaluated, as in the reproducibility analysis, a cutoff value of 0.15 was chosen for the percent absolute difference, that is, the difference with respect to the baseline images. The robustness varied from 97.02% (456 of 470 CTTA metrics) when the field of view was changed to 8.93% (105 of 1175 CTTA metrics) when the slice thickness was varied on the Philips scanner. The difference in the number of CTTA variables stems from the fact that more combinations were tested some variables compared to the others (Table 1). For example, five combinations of slice thickness were considered, so 5 × 235 texture metrics = 1175 CTTA metrics were considered. Similarly, only two combinations of field of view were considered, so 2 × 235 texture metrics = 470 CTTA metrics were considered. Robustness measures dropped drastically on the Toshiba scanner, the values ranged from 3.19% (15 of 470 CTTA metrics) when the noise filters were used to 9.57% (45 of 470 CTTA metrics) when the field of view was changed.

In general, FFT metrics show good robustness (blue bands in the robustness map next to FFT, Metric 218 220 224 and 225). In general, texture metrics are more robust on the Philips scanner compared to the Toshiba scanner.

### 3.3 Reliability

By assessing the consistency of robust and repeatable CTTA metrics across the two scanners, we see that only select FFT metrics (Metric 218 220 224 and 225) show strong signal on both scanners and hence most reliable at least across the two scanners in consideration. The reliable FFT metrics are related to assessing entropy of the FFT magnitude and phase.

### 3.4 Effect of post‐processing techniques that reduce image noise while preserving the underlying edges associated with true anatomy or pathology

**Figure 3:**
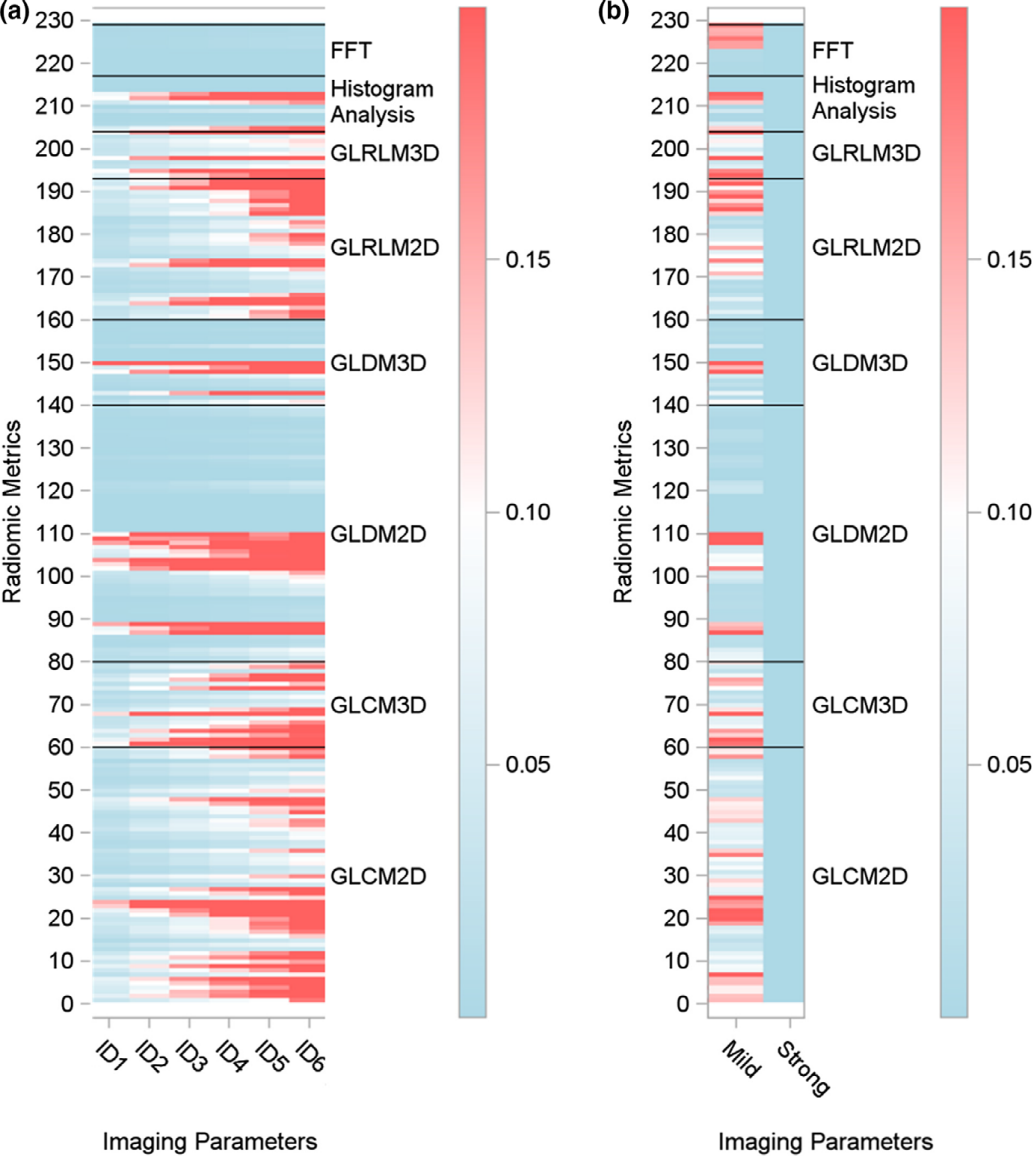
Robustness assessment of the texture metrics due to changes in reconstruction filters; I‐dose levels (Philips scanner [a] and changes in noise corrections levels (Mild or Strong) on the Toshiba scanner[b].

By comparing the changes in robustness of the CTTA metrics across the two scanners, we observe that post‐processing techniques that reduce image noise while preserving the underlying anatomical edges for example, I‐dose levels (here 6 levels) on the Philips scanner and Mild/Strong (here 2 levels) levels on the Toshiba scanner produce significant difference in CTTA robustness compared to the base setting (Fig. 3). Stronger noise reduction techniques were associated with a significant reduction in reliability in the Philips scanner, however, the opposite was observed on the Toshiba scanner. In both cases, no noise reduction techniques were used in the base setting.

## 4 DISCUSSION

While CTTA based tumor‐modeling assessment is increasingly being reported,^1^, ^2^, ^3^, ^4^, ^5^, ^6^, ^7^ a consensus on radiomics reliability has not emerged leading to an increased risk of false discovery. Such a scenario can impede the clinical translation of radiomics. The primary objective of our study was to identify CTTA metrics that are reliable using a CT‐based texture phantom. We specifically assess the intra‐, inter‐ and test‐retest variations of 235 CTTA metrics derived from eight different texture methods using the texture phantom imaged under different CT imaging settings.

Several studies have been conducted to investigate the temporal stability of CTTA metrics. However, most of these studies have been limited to performing the stability assessment using the Credence Cartridge Radiomics (CCR) Phantom in a test‐retest setting and for different combinations of scanners, imaging, and post‐processing protocols.^10^, ^11^, ^24^, ^25^ By design, the CCR phantom has been customized to assess the reliability of global texture metrics assessed from non‐small cell lung carcinoma (NSCLC) imaging data. Studies by Mackin et al reporting the first use of the CCR phantom established that the inter‐scanner variability of the radiomic metrics depended on both the cartridge material of the CCR phantom and the metric being assessed.^10^ While the range of the scan acquisition parameter variations was limited to those used in imaging NSCLC patients, the study identified select radiomics metrics extracted from NSCLC tumors that had large inter‐scanner variability compared to the inter‐patient variability. Yet another limitation of the phantom is its cuboidal shape which makes it prone to CT‐based imaging artefacts and also, only a couple of cartridge inserts, namely rubber and acrylic have been further analyzed in other studies.^24^, ^25^ Our phantom is an improvement over the anthropomorphic thorax phantom used by Zhao et al., as our ROIs were designed to be heterogeneous in density to evaluate image textures.^26^ Future versions of our phantom will consider using the 3D shapes as suggested by Zhao et al. in combination with our texture patterns.

From a material standpoint, our texture phantom was made using ABS plastic using 3D printing technologies and casting them into tissue density urethane. Our intention was to create a generic phantom that could be imaged using diverse imaging protocols and scanners to identify reliable CTTA metrics. We focused on using materials and designs inspired from a wide span of tumor textures seen in oncological CT images. Subsequently, the materials selected for our tests were within the tissue density and texture range that provided targeted contrast within our texture patterns. This is an improvement over texture analysis reliability studies conducted using a water phantom.^27^ Last, but not least, from a design standpoint, our texture phantom is designed as a short length cylinder, similar to the standard ACR/AAPM phantom^28^ which is comparatively more CT‐friendly design in terms of reducing imaging artefacts compared to a cuboid.

From an analysis standpoint, using the concordance correlation coefficient (CCC) as a metric for stability, a few studies assess the variation in radiomic metric values with changes in slice thickness, exposure and bin width. Studies by Larue et al., showed that of these three, slice thickness and exposure were dominant. Changes due to slice thickness could be overcome by resampling, however, no clear relationship between radiomic metrics and exposure was established.^25^ While the finding of this study is important, some of the important limitations of this study include that study was limited to the rubber insert of the CCR phantom, which was identified in prior studies by Mackin et al to produce the most comparable radiomic metrics distribution comparable to NSCLC datasets. Therefore, the findings are not translatable to other tumor types. Most recent studies conducted by Mackin et al., indicate that variable x‐ray tube current is unlikely to have a large effect on radiomic features extracted from computed tomography images of textured objects such as tumors.^10^ Studies by Hassan et al., showed that normalizing voxel size and gray‐level discretization greatly reduced the dependence of CTTA metrics on these quantities.^24^ While novel CTTA metrics such as first‐order wavelet features were assessed in this reliability study, the limited suitability of the CCR phantom to truly assess the performance of metrics of local texture variations limits the scope of these findings. Recent studies by Lu et al., using the a Gammex CT ACR 464 phantom^29^ and scanning its four water equivalent inserts using routine abdomen protocols on a GE Discovery scanner reported that highly reliable radiomic metrics were attained from images reconstructed at high tube current and thick slice thickness. Also, based on a ranking of the reliability of commonly used CTTA metrics, first‐order texture metrics such as mean, standard deviation, skewness, kurtosis were more reliable than second‐order texture metrics such as GLCM‐energy, correlation, contrast, and homogeneity. While encouraging, the study was tested on only one scanner and using two materials for each pattern.

In addition to designing a more sophisticated phantom that mimic in vivo lesions with the help of 3D‐printing technique, we include a new addition, that is, FFT‐based features in this analysis. Considering that FFT‐based metrics still assess the global variations in texture, it is a valuable addition to the CTTA panel and suitable for evaluation using our phantom and the CCR phantom. While the reliability of FFT‐based metrics are being assessed for the first time, its predictive value has been assessed in prior studies. Published literature shows that FFT‐based spectral metrics can differentiate between ccRCC grades and differentiating solid, non‐macroscopic fat containing, enhancing renal masses based on their CT images.^21^ While using wavelet features that have also shown great potential in characterizing tumor behavior^15^, ^30^ would have been a more generalized approach to FFT, a phantom made of multi‐material would be best suited to study the local variations in texture captured by the wavelet metrics and assess its reliability. Our texture pattern is based on 3D printing a single material, which is immersed a casting material. While the boundaries serve as multi‐material regions, the assessment will be limited and unrealistic owing to simplification. Our next generation/ version of the texture phantom will include this.

Identification of reliable CTTA metrics is an important step toward the clinical translation of radiomics. Our approach of identifying such metrics differs from studies in the literature^13^, ^31^ by the use of a new texture phantom and a rigid ROI registration, which helps in eliminating the reported challenges such as patient‐based tumor variability and segmentation bias/errors. In addition, we assessed all the CT acquisition parameters together, which to the best of our knowledge has not been performed before. Finally, we assessed the reliability of CTTA metrics by using comparable acquisition protocols, over a wide range of values, over two CT scanners. Similar studies^9^, ^10^ have addressed such issues but their use of automatic acquisition protocols for different CT scanners, lead to reproducibility concerns, particularly since, automatic acquisition protocols optimize imaging parameters such as tube current, slice thickness etc., which make it impossible to study the effects of various imaging parameters simultaneously at the same time.

Different CT scanners have been proven to report a variation in Hounsfield units.^10^ In addition to scanner specific calibration, these differences may be responsible for the changes in radiomics reproducibility across the two scanners. Our results support this fact and identify reliable CTTA metrics for use across different scanners. In addition to testing the reliability of CTTA metrics to image acquisition parameters, also tested its reliability to changes brought about by using noise reduction techniques such as I‐dose etc. As expected, due its nonlinear effect, the use of I‐dose levels affects radiomic reliability significantly. The CTTA reliability reduces with the increase in I‐dose levels.

Published data assessing radiomics reproducibility and repeatability have reported a high reliability is associated with the entropy measure of first‐order statistical measure (e.g., histogram analysis).^32^ Though we do not observe histogram‐based entropy measure to be reliable in our experiments, we do observe entropy of FFT magnitude and FFT phase to be reliable.^16^ While other measures of local variation such as GLRLM failed to show reliability, entropy of FFT‐based metrics such as magnitude and phase which also assess local variations of texture showed reliability. The reduced sensitivity of the FFT measures to the scanner and imaging confounds needs to be further evaluated. However, within the limitation of the study, the entropy of FFT‐based magnitude and phase was shown to be more reliable than other texture metrics across the two scanners and imaging conditions we considered.

In our study, we observe that nonlinear noise reduction techniques such as I‐dose levels bring about a significant difference in the robustness of the CTTA metrics compared to the when such techniques were not used. Future studies assessing the effects of such post‐reconstruction noise reduction techniques are warranted.

One of the limitations of our study is that we used a texture phantom; therefore, a biological correlation for identified CTTA metrics could not be addressed. However, development of texture phantoms is crucial to conducting inter‐scanner, intra‐scanner, and test‐retest variability of CTTA metrics within a multicenter setting. Once reliable CTTA metrics are identified follow‐up studies assessing biological correlation can be designed. Another limitation of our study is that our result could be affected by the fundamental design differences of the scanners. Rather than following clinical protocol, we decided to use imaging‐variables that spanned the values seen in clinical scans. This approach allowed us to assess the variability in the phantom scans as a result of characteristics inherent to the scanner. Using our approach, imaging protocols that produce high‐reliability CTTA metrics can be identified, however, this is not the focus of the paper. Further, our work was limited to only two scanners and the effect of factors such as motion, scatter, noise etc. was not assessed. In the developed texture phantom, we use textures nonspecific to a given human tissue, but this was done to improve the comprehensive assessment of a variety of human tissue textures than that of a specific one.

The two commonly used statistical indices to assess reliability include the intraclass correlation coefficient (ICC) and the CCC.^33^ When assessing reproducibility alone without repeating multiple times for a given scanner or modality, the ICC2 (two‐way random) and ICC3 (two‐way mixed) are identical to CCC. However, if with repeated measures, which is equivalent to assessing reproducibility and repeatability at once, only ICC3 (two‐way mixed) is identical to CCC. CCC or ICC2/ICC3 include two components for claiming reliable (a) small difference between measurements (b) correlated result between measurements. An excellent CCC or ICC will represent both the small difference and high correlation, however when the CCC or ICC value is moderate, it will be hard to pin point whether the problem is from large difference or poor correlation. In this preliminary study, we only have three inserts for the phantom, thus we are only interested in observing the signal change (difference) when altering the scanner settings, or between scanners. The absolute percent difference is very intuitive to serve the purpose of this study. If evidence established for reliability in signal difference, we will proceed further study with more heterogeneous inserts (e.g., 9) and investigate both difference and correlation.

Various studies have shown the valuable role of CTTA metrics in tumor characterization, prognosis, and survival information, albeit using a small sample size.^6^, ^21^, ^33^, ^34^, ^35^ While The Cancer Imaging Archive (TCIA) database can aid large‐scale validation of the CTTA panel, additional problems such as noisy or missing data can reduce the impact. Machine‐learning methods have been used to augment these limitations,^36^ however, the choice of machine‐learning algorithms and associated steps affects the final performance and thus far a consensus has not been reached. Future work within our group will evaluate the clinical applications of our results using data‐driven radiomics^37^ frameworks in combination with TCIA data.

In conclusion, our study has demonstrated the intra‐, inter‐ and test‐retest variation in CTTA metrics calculated on CT images of a texture phantom imaged using two different CT scanners. We identify reliable CTTA metrics, that is, those metrics with less < 5% change in its value when assessing for robustness, reproducibility, and repeatability. We strongly recommend that groups working on future radiomic studies account for the performance variations demonstrated here and/ or use the reliable CTTA metrics, that is, Entropy of FFT‐based magnitude and phase, within their radiomics texture panel.

## CONFLICT OF INTEREST

No conflicts of interest.

## Supporting information

Supinfo. CTTA metrics used in the reliability assessment heatmap.
