# Supplementary material for: Reliability of CT‐based texture features: Phantom study
Source: J Appl Clin Med Phys. 2019 Jun 20;20(8):155–63. doi: 10.1002/acm2.12666 (PMC6698768; doi:10.1002/acm2.12666)
Supplement: Supplementary file 1 — Supinfo. CTTA metrics used in the reliability assessment heatmap. [file ACM2-20-155-s001.pdf]

Supplementary S1: CTTA metrics used in the reliability assessment heatmap

Additional details regarding equations etc. can be found as supplementary information in the following manuscript: *Varghese B, Chen F, Hwang D, Palmer SL, De Castro Abreu AL, Ukimura O, et al. Objective risk stratification of prostate cancer using machine learning and radiomics applied to multiparametric magnetic resonance images. Sci Rep. 2019 Feb 7;9(1):1570*

Texture Method	References	CTTA metric		Position
Greylevel co-occurrence matrix (GLCM 2D) (60 metrics)	Haralick RM (1979) Statistical and structural approaches to texture. Proceedings of the IEEE 67: 786–804. Haralick RM, Shanmugam K, Dinstein I (1973) Textural features for image classification. IEEE Transactions on Systems, Man, and Cybernetics 3: 610–621.	Angular Second Moment	ASM_Ax	1
			ASM_Cor	2
			ASM_Sag	3
		Contrast	CON_Ax	4
			CON_Cor	5
			CON_Sag	6
		Correlation	Correlation_Ax	7
			Correlation_Cor	8
			Correlation_Sag	9
		Dissimilarity	DIS_Ax	10
			DIS_Cor	11
			DIS_Sag	12
		Entropy	ENT_Ax	13
			ENT_Cor	14
			ENT_Sag	15
		Homogeneity	HOM_Ax	16
			HOM_Cor	17
			HOM_Sag	18
		Inverse Difference Moment	IDM_Ax	19
			IDM_Cor	20

			IDM_Sag	21
		Informational Measure of Correlation 1	IMC1_Ax	22
			IMC1_Cor	23
			IMC1_Sag	24
		Informational Measure of Correlation 2	IMC2_Ax	25
			IMC2_Cor	26
			IMC2_Sag	27
		Maximal Correlation Coefficient	MCC_Ax	28
			MCC_Cor	29
			MCC_Sag	30
		Mean	Mean_Ax	31
			Mean_Cor	32
			Mean_Sag	33
		Square root of Variance	SQV_Ax	34
			SQV_Cor	35
			SQV_Sag	36
		Standard Deviation	StdDev_Ax	37
			StdDev_Cor	38
			StdDev_Sag	39
		Uniformity	Uniformity_Ax	40
			Uniformity_Cor	41
			Uniformity_Sag	42
		Variance	Var_Ax	43
			Var_Cor	44

			Var_Sag	45
		Difference of mean	difAve_Ax	46
			difAve_Cor	47
			difAve_Sag	48
		Difference of entropy	difENT_Ax	49
			difENT_Cor	50
			difENT_Sag	51
		Sum of averages	sumAve_Ax	52
			sumAve_Cor	53
			sumAve_Sag	54
		Sum of entropy	sumENT_Ax	55
			sumENT_Cor	56
			sumENT_Sag	57
		Sum of variance	sumVar_Ax	58
			sumVar_Cor	59
			sumVar_Sag	60
Greylevel co-occurrence matrix (GLCM 3D) (20 metrics)		Angular Second Moment	ASM_3D	61
		Contrast	CON_3D	62
		Correlation	Correlation_3D	63
		Dissimilarity	DIS_3D	64
		Entropy	ENT_3D	65
		Homogeneity	HOM_3D	66
		Inverse Difference Moment	IDM_3D	67
		Informational Measure of Correlation 1	IMC1_3D	68

		Informational Measure of Correlation 2	IMC2_3D	69
		Maximal Correlation Coefficient	MCC_3D	70
		Mean	Mean_3D	71
		Square root of Variance	SQV_3D	72
		Standard Deviation	StdDev_3D	73
		Uniformity	Uniformity_3D	74
		Variance	Var_3D	75
		Difference of mean	difAve_3D	76
		Difference of entropy	difENT_3D	77
		Sum of averages	sumAve_3D	78
		Sum of entropy	sumENT_3D	79
		Sum of variance	sumVar_3D	80
Greylevel difference matrix (GLDM 2D) (60 metrics)	Haralick RM (1979) Statistical and structural approaches to texture. Proceedings of the IEEE 67: 786–804. Haralick RM, Shanmugam K, Dinstein I (1973) Textural features for image classification. IEEE Transactions on Systems, Man, and Cybernetics 3: 610–621.	Angular Second Moment	ASM_Ax	81
			ASM_Cor	82
			ASM_Sag	83
		Contrast	CON_Ax	84
			CON_Cor	85
			CON_Sag	86
		Correlation	Correlation_Ax	87
			Correlation_Cor	88
			Correlation_Sag	89
		Dissimilarity	DIS_Ax	90
			DIS_Cor	91
			DIS_Sag	92

		Entropy	ENT_Ax	93
			ENT_Cor	94
			ENT_Sag	95
		Homogeneity	HOM_Ax	96
			HOM_Cor	97
			HOM_Sag	98
		Inverse Difference Moment	IDM_Ax	99
			IDM_Cor	100
			IDM_Sag	101
		Informational Measure of Correlation 1	IMC1_Ax	102
			IMC1_Cor	103
			IMC1_Sag	104
		Informational Measure of Correlation 2	IMC2_Ax	105
			IMC2_Cor	106
			IMC2_Sag	107
		Maximal Correlation Coefficient	MCC_Ax	108
			MCC_Cor	109
			MCC_Sag	110
		Mean	Mean_Ax	111
			Mean_Cor	112
			Mean_Sag	113
		Square root of Variance	SQV_Ax	114
			SQV_Cor	115
			SQV_Sag	116

		Standard Deviation	StdDev_Ax	117
			StdDev_Cor	118
			StdDev_Sag	119
		Uniformity	Uniformity_Ax	120
			Uniformity_Cor	121
			Uniformity_Sag	122
		Variance	Var_Ax	123
			Var_Cor	124
			Var_Sag	125
		Difference of mean	difAve_Ax	126
			difAve_Cor	127
			difAve_Sag	128
		Difference of entropy	difENT_Ax	129
			difENT_Cor	130
			difENT_Sag	131
		Sum of averages	sumAve_Ax	132
			sumAve_Cor	133
			sumAve_Sag	134
		Sum of entropy	sumENT_Ax	135
			sumENT_Cor	136
			sumENT_Sag	137
		Sum of variance	sumVar_Ax	138
			sumVar_Cor	139
			sumVar_Sag	140

Greylevel difference matrix (GLDM 3D) (20 metrics)		Angular Second Moment	ASM_3D	141
		Contrast	CON_3D	142
		Correlation	Correlation_3D	143
		Dissimilarity	DIS_3D	144
		Entropy	ENT_3D	145
		Homogeneity	HOM_3D	146
		Inverse Difference Moment	IDM_3D	147
		Informational Measure of Correlation 1	IMC1_3D	148
		Informational Measure of Correlation 2	IMC2_3D	149
		Maximal Correlation Coefficient	MCC_3D	150
		Mean	Mean_3D	151
		Square root of Variance	SQV_3D	152
		Standard Deviation	StdDev_3D	153
		Uniformity	Uniformity_3D	154
		Variance	Var_3D	155
		Difference of mean	difAve_3D	156
		Difference of entropy	difENT_3D	157
		Sum of averages	sumAve_3D	158
		Sum of entropy	sumENT_3D	159
		Sum of variance	sumVar_3D	160
Greylevel run-length matrix (GLRLM 2D) (33 metrics)	Tang X. Texture information in run-length matrices. IEEE transactions on image processing. 1998 Nov;7(11):1602-9.	Gray Level Non-Uniformity	GLN_Ax	161
			GLN_Cor	162
			GLN_Sag	163
		High gray-level emphasis	HGRE_Ax	164

			HGRE_Cor	165
			HGRE_Sag	166
		Low gray-level emphasis	LGRE_Ax	167
			LGRE_Cor	168
			LGRE_Sag	169
		Long-run emphasis	LRE_Ax	170
			LRE_Cor	171
			LRE_Sag	172
		Long-run, high gray-level emphasis	LRHGE_Ax	173
			LRHGE_Cor	174
			LRHGE_Sag	175
		Long-run, low gray-level emphasis	LRLGE_Ax	176
			LRLGE_Cor	177
			LRLGE_Sag	178
		Run length nonuniformity	RLN_Ax	179
			RLN_Cor	180
			RLN_Sag	181
		Run percentage	RP_Ax	182
			RP_Cor	183
			RP_Sag	184
		Short-run, low gray-level emphasis	SGLGE_Ax	185
			SGLGE_Cor	186
			SGLGE_Sag	187
		Short-run emphasis	SRE_Ax	188

			SRE_Cor	189
			SRE_Sag	190
		Short-run, high gray-level emphasis	SRHGE_Ax	191
			SRHGE_Cor	192
			SRHGE_Sag	193
Greylevel run-length matrix (GLRLM 3D) (11 metrics)		Gray Level Non-Uniformity	GLN_3D	194
		High gray-level emphasis	HGRE_3D	195
		Low gray-level emphasis	LGRE_3D	196
		Long-run emphasis	LRE_3D	197
		Long-run, high gray-level emphasis	LRHGE_3D	198
		Long-run, low gray-level emphasis	LRLGE_3D	199
		Run length nonuniformity	RLN_3D	200
		Run percentage	RP_3D	201
		Short-run, low gray-level emphasis	SGLGE_3D	202
		Short-run emphasis	SRE_3D	203
		Short-run, high gray-level emphasis	SRHGE_3D	204
Histogram analysis (Intensity) (13 metrics)	Haralick RM (1979) Statistical and structural approaches to texture. Proceedings of the IEEE 67: 786–804.	Kurtosis	Kurtosis_3D	205
		Maximum	Max_3D	206
		Mean	Mean_3D	207
		Median	Median_3D	208
		Minimum	Min_3D	209
		No.of voxels	N_3D	210
		Standard Deviation	STD_3D	211
		Skewness	Skewness_3D	212

		Variance	Variance_3D	213
		Volume	Volume_3D	214
		Volume voxels (X)	VoxelDimX_3D	215
		Volume voxels (Y)	VoxelDimY_3D	216
		Volume voxels (Z)	VoxelDimZ_3D	217
Fast Fourier Transform analysis (FFT) (18 metrics)	Varghese BA, Chen F, Hwang DH, Cen SY, Gill IS, Duddalwar VA. Differentiating solid, non-macroscopic fat containing, enhancing renal masses using fast Fourier transform analysis of multiphase CT. Br J Radiol. 2018 Jun 21;20170789.	Entropy of FFT magnitude (10 to 90% filter)	EVMag1_Ax	218
			EVMag1_Cor	219
			EVMag1_Sag	220
		Entropy of FFT magnitude (no filter)	EVMag_Ax	221
			EVMag_Cor	222
			EVMag_Sag	223
		Complexity Index (10 to 90% filter)	SumFreq2_1_Ax	224
			SumFreq2_1_Cor	225
			SumFreq2_1_Sag	226
		Complexity Index (no filter)	SumFreq2_Ax	227
			SumFreq2_Cor	228
			SumFreq2_Sag	229
		Entropy of FFT phase (10 to 90% filter)	EVPhase1_Ax	230
			EVPhase1_Cor	231
			EVPhase1_Sag	232
		Entropy of FFT phase (no filter)	EVPhase_Ax	233
			EVPhase_Cor	234
			EVPhase_Sag	235
